# Grief Instruments in Children and Adolescents: A Systematic Review

**DOI:** 10.1177/00302228231171188

**Published:** 2023-04-20

**Authors:** Toni Zhang, Karolina Krysinska, Eva Alisic, Karl Andriessen

**Affiliations:** 1 Melbourne Medical School, 2281 The University of Melbourne, Melbourne, VIC, Australia; 2 Melbourne School of Population and Global Health, 2281 The University of Melbourne, Melbourne, VIC, Australia

**Keywords:** bereavement, assessment, inventory, psychometrics, youth

## Abstract

Many children and adolescents experience the death of a close person, such as a family member or a friend. However, there is a scarcity of literature on the assessment of grief in bereaved youth. The use of validated instruments is essential to advance our knowledge of grief in children and adolescents. We conducted a systematic review, adhering to PRISMA guidelines, to identify instruments that measure grief in this population and explore their characteristics. Searches in six databases (Medline, PsycINFO, Embase, Emcare, Scopus, and Web of Science) identified 24 instruments, encompassing three categories: general-purpose grief scales, maladaptive grief scales, and specialized grief scales. We extracted data using a predetermined list of descriptive and psychometric properties. Findings indicate a need to direct research towards more stringent validation of existing instruments and the design of new instruments in line with developments in the understanding of grief in this population.

## Introduction

Many children and adolescents experience the death of a family member or friend. Approximately 4% of adolescents have experienced the death of a parent, approximately 1.5% have lost a sibling, and one in five adolescents have experienced the death of a close friend by the age of 18 (Hulsey et al., 2020; Rheingold et al., 2004). A nationwide study in Scotland reported that 51% of the children had lost a close family member by age 8, with a higher ratio in children from low-income households (Paul & Vaswani, 2020). Moreover, it has been estimated that over 140,000 children have experienced the death of a caregiver due to COVID-19 in the US (Hillis et al., 2021).

The death of a loved one is a distressing and disruptive event in the lives of children and adolescents. Grief is defined as the natural and primarily affective reaction to such loss, encompassing diverse psychological (emotional and cognitive), somatic, and behavioural responses (Stroebe et al., 2008). Common grief reactions in children and adolescents include crying, feelings of sadness, anger, guilt, and longing to be reunited with the deceased person. They may also experience difficulties with concentration, sleeping, or school performance (Balk, 2014; Elsner et al., 2022). They can become socially withdrawn or develop health risk behaviours such as fighting or substance use (Andriessen et al., 2018a).

Despite the challenges of bereavement, most young people tend to cope well with their grief, and some may experience positive transformations, such as posttraumatic or personal growth, because of their struggles with the aftermath of the bereavement (Salloum et al., 2019). Bereaved adolescents have described this phenomenon as ‘life lessons’ that result in lasting positive changes in their perception of self, relationships, or life, which can be experienced through increased self-care, empathy for and sense of closeness with others, and engaging in new possibilities (Andriessen et al., 2018b). Though further research is needed, it seems that being able to share bereavement experiences, receiving social support, and positive experiences regarding continuing bonds with the deceased, contribute to developing personal growth in this population (Levi-Belz et al., 2021; Sharp et al., 2018; Tan & Andriessen 2021).

Nonetheless, research in this field has been focused mostly on negative grief reactions and risks for long-term adverse outcomes (Feigelman et al., 2017), which may be due to pre-existing personal or familial mental health or relational problems, and diminished quality of relationships after the loss (Andriessen et al., 2016; Stikkelbroek et al., 2016). Bereaved children and adolescents have a 2.5 times higher risk of long-term mental health problems such as anxiety and depression, especially after a traumatic death such as suicide, compared to non-bereaved youth (Burrell et al., 2022). Studies have reported increased risk of depression in about 10% of adolescents bereaved by a suicide or other traumatic death of a parent more than 2 years after the loss (Melhem et al., 2011). Bereaved children and adolescents are at a threefold increased risk of dying by suicide (Wilcox et al., 2010), and death of a parent has been associated with an increased risk of premature death and all-cause mortality (Feigelman et al., 2017; Li et al., 2014).

Over the decades bereavement research has examined maladaptive or adverse grief reactions, and differentiated it from other forms of psychological distress, such as depression and posttraumatic stress (Lundorff et al., 2017; Prigerson et al., 2021). Also specifically in bereaved children and adolescents, adverse grief is associated with a differentiable set of reactions that independently increase the risk of functional impairment (Geronazzo-Alman et al., 2019; Spuij et al., 2012b). The inclusion of Prolonged Grief Disorder (PGD) as a separate diagnostic entity in the Diagnostic and Statistical Manual of Mental Disorders (5^th^ Edition; DSM-V-TR; American Psychiatric Association, 2020) and International Classification of Diseases (11^th^ Revision; ICD-11; World Health Organisation, 2019) highlights the importance of detecting maladaptive grief reactions that confer a higher vulnerability to depression and significant functional impairment in this population (Boelen et al., 2019).

Instruments that measure grief are imperative to enhance our understanding of natural grief reactions, maladaptive responses to grief and the needs of bereaved youth, as well as enabling the prescription of appropriate treatments and assessment of outcomes following intervention. Appropriate grief assessment tools may also help elucidate risk factors for the development of mental health problems later in life, including suicidal ideation (Hill et al., 2019). In children and adolescents in particular, developmental influences and differential manifestations of grief are necessary to consider in the assessment of grief reactions (Kaplow et al., 2012). However, a dearth of well-validated instruments has hindered the ability to explore natural and adverse responses to grief in this population (Andriessen et al., 2021; Kentor & Kaplow, 2020). Whilst research is increasing in this field, a review of the available instruments for assessing grief in children and adolescents is yet to be published. Addressing this gap in the literature, this study aimed to identify the available instruments for assessing grief in children and adolescents and explore their characteristics. A summary of the characteristics and psychometric properties is provided to present a resource that can be utilized in the clinical or research setting to assist in the evaluation of bereaved youth.

## Methods

### Search Strategy

Based on the PRISMA guidelines (Page et al., 2021), the review involved searches in Medline, PsycINFO, Embase, Emcare (all accessed via Ovid), Scopus, and Web of Science. Search terms for Ovid databases were (exp Grief/OR grief.ti,ab. OR exp Bereavement/OR bereve*.ti,ab.) AND (instrument* OR tool* OR inventory OR inventories OR measure* OR assessment OR scale). ti,ab. AND (adolescent*.ti,ab. OR child*.ti,ab. OR youth.ti,ab.). Search terms for Scopus and Web of Science databases were (grief OR bereave*) AND (instrument* OR tool* OR inventory OR inventories OR measurement* OR assessment OR scale*) AND (adolescen* OR child* OR youth), with filter for title and abstract only applied. Researcher T.Z. conducted the searches in May 2022, and updated the searches in December 2022. The searches were limited to peer-reviewed publications in English, but not by location or year of publication.

**Figure 1.**
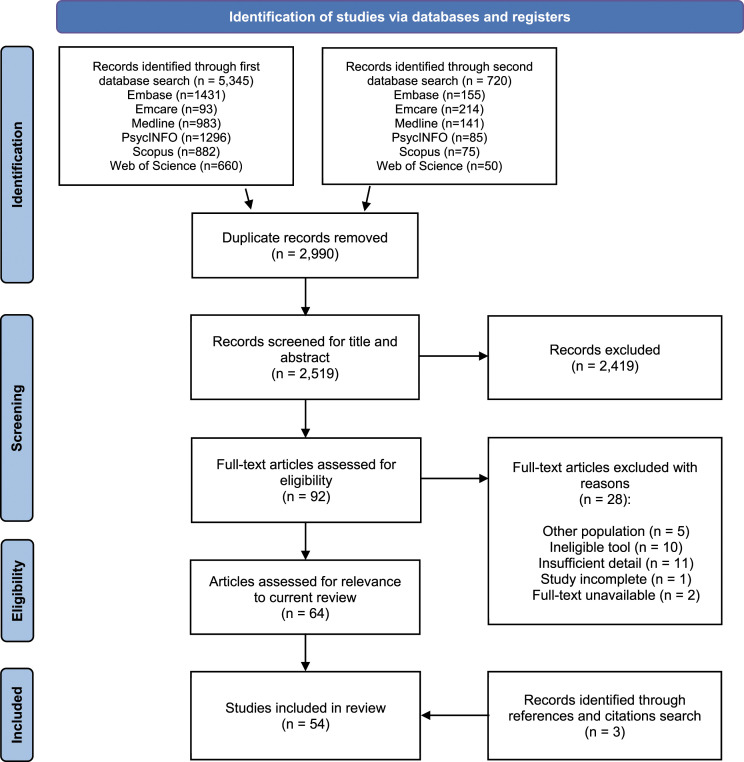
PRISMA flow diagram.

Researcher T.Z. removed the duplicates and two researchers (T.Z. and K.A.) independently screened titles and abstracts for eligibility, followed by assessment of full texts against the inclusion and exclusion criteria. Disagreements were resolved by team discussion. Where multiple articles were available for a particular instrument, only articles that provided psychometric data were included in the review. Those that contained solely descriptive information about instruments without psychometric analysis were excluded. The references of the selected studies as well as a forward citation search was performed with the selected studies to identify additional instruments or descriptive or psychometric information relevant to the current study. Figure 1 presents the search and selection process.

### Inclusion and Exclusion Criteria

Studies were included if: (1) the study population consisted of children or adolescents up to 18 years of age, (2) the study involved the development, use and/or evaluation of a standardized grief instrument for children and adolescents, (3) the study was published in a peer-reviewed journal, in English or English translation was available.

Studies were excluded if: (1) the study population primarily consisted of individuals over 18 years of age, including the use of an assessment tool on young adults or adults who had experienced bereavement as a child or adolescent, (2) the study investigated non-human bereavement (e.g., pets), (3) the publication was a book review, editorial, dissertation, or other non-peer-reviewed publication.

### Data Extraction

We collated the grief instruments and created a data extraction form based on the variables included in the COSMIN Risk of Bias tool (Mokkink et al., 2020). The COSMIN (COnsensus-based Standards for the selection of health Measurement Instruments) tool outlines the standards and psychometric variables used to assess the quality of studies and outcome measurement instruments (Mokkink et al., 2020). Psychometric variables outlined in the COSMIN tool that were minimally represented in the articles were excluded from the data extraction process. The following predetermined list was developed to guide data extraction:

• Instrument and article details: name of the instrument and the author and year of development. Where the origin paper of the instrument was not included in the search results, it was sought through citations from the studies identified in the search and referenced in the tables.
• Target population details: age range, children and/or adolescents, and the type of bereavement. Where the intended age range was not explicitly stated, it was inferred from the sample on which the validation study was undertaken.
• Practical information: languages available, measurement properties including mode of report and scale used.
• Instrument structure: number of items, factor structure or subscales.
• Psychometric information: reliability (internal consistency and test-retest correlation) and validity (concurrent validity, convergent validity and divergent validity).

In case conflicting information was present regarding a single instrument being used in multiple studies, the information provided in the most recent study was reported.

## Results

The systematic review identified 24 instruments for the assessment of grief in children and adolescents, developed between 1987 and 2022. These instruments can be classified into three broad categories in relation to the types of grief reactions being assessed: general-purpose grief scales (*n* = 9), instruments assessing maladaptive grief reactions (*n* = 13), and specialized grief scales (*n* = 2). While previous discussion of grief instruments for adult populations distinguished between general-purpose and specialized grief instruments (i.e., for selected populations) (Neimeyer et al., 2008), the current study recognizes maladaptive grief reaction measures as a separate classification entity. The descriptive and psychometric properties of the instruments have been provided in Tables 1 and 2, respectively.

**Table 1.** Summary of the instruments and their descriptive characteristics.

Instrument	Sample Characteristics	Aims	Measurement Properties	Demographics	Languages	Number of Items	Factor Structure
Adolescent grief inventory (AGI)	12 to 18-year-olds who experienced the loss of a family member or friend through any cause	To assess grief in bereaved adolescents	Self-report	Validated in a sample of Australian adolescents (*n* = 176), majority Caucasian (72.2%), female (80.6%) and studying or employed (72.7%) (Andriessen et al., 2018b)	English	40	Six-factor structure
(Andriessen et al., 2018a)			5-Point likert scale from 1 “not at all” to 5 “extremely”		Translated into Dutch (college student samples)		1. Sadness (11 items)
							2. Self-blame (5 items)
							3. Anxiety and self-harm (8 items)
							4. Shock (4 items)
							5. Anger and betrayal (5 items)
							6. Sense of peace (7 items)
							(Andriessen et al., 2018a)
Adolescent grief response inventory (AGRI)	Adolescents who have experienced the loss of a loved one	To assess the adolescent’s functioning during their experience of grief and their individual grief needs to better tailor interventions and treatments	7-Point likert scale from 1 “completely disagree” to 7 “completely agree”	Validated in a sample of public high school students in the US (*n* = 250), majority Caucasian (Petscher, 2006)	English	34	Five-factor structure (based on the five components of wellness)
(Petscher, 2006)							1. Psychological (8 items)
							2. Emotional (6 items)
							3. Social (6 items)
							4. Spiritual (7 items)
							5. Physical (8 items)
							(Petscher, 2006)
Bereaved cancer needs instrument (BCNI)	Adolescents and young adults aged 12–25 years of age who have experienced the death of a parent or sibling due to cancer	To assess the unmet psychosocial needs of adolescents and young adults bereaved by cancer in order to provide appropriate support	4-Point scale	Validated in a sample of Australian youth (*n* = 335), 65.5% female, with a family member lost to cancer (Patterson et al., 2020)	English	37	Seven-factor structure
(Patterson et al., 2020)							1. Help and information about grief
							2. Time-out from grief
							3. Planning for the future
							4. Support from friends
							5. Talk to others with similar experiences
							6. Dealing with feelings
							7. Family connectedness (Patterson et al., 2020)
Extended grief inventory (EGI)	Children and adolescents aged between 6 and 18 who have experienced the death of a parent, sibling or other loved one	To measure the frequency of grief reactions and assess adaptive and maladaptive grief responses in bereaved children and adolescents	Self-report	Validity information not available	English	28 (McClatchey et al., 2014)	Three-factor structure (Brown & Goodman, 2005)
(Layne et al., 2001)			0–100 point scale. (Dalton & Krout, 2005)		Translated into Khmer (Field et al., 2014)		1. Traumatic grief (23 items)
					Translated to Arabic (Morgos et al., 2008)		2. Positive memory (3 items)
							3. Ongoing presence (2 items)
							Three-factor structure (Layne et al., 2001, cited in McClatchey et al., 2014)
							1. Positive connection (5 items)
							2. Existentially complicated grief reactions (8 items)
							3. Traumatic intrusion and avoidance (11 items)
Grief cognitions questionnaire for children (GCQ-C)	8 to 18-year-old children and adolescents who have experienced the death of a family member	To examine the negative cognitions associated with prolonged grief disorder and other forms of emotional distress following bereavement of a loved one	Self-report, parent-report	Validated in a sample of Dutch children (*n* = 151) (Spuij et al., 2017)	Dutch	20	One-factor structure – “overall loss-related negative thinking” (Spuij et al., 2017)
(Spuij et al., 2017)			3-Point likert scale: 0 (hardly ever); 1 (sometimes); 2 (always)		English translation available		
Grief facilitation inventory (GFI)	6 to 18-year-old children and adolescents who have reported experiencing a death	To measure caregiver behaviours that influence grief reactions in children	Self-report, caregiver-report	Validated in a sample of youth of hispanic/Latinx (46%), Caucasian (23%), black (13%) and mixed/biracial (13%) ethnicity (*n* = 432) (Alvis et al., 2022b)	English	24	Four-factor structure
(Alvis et al., 2022b)			5-Point frequency scale: 0 (not at all) to 4 (all the time) to assess the frequency of caregiver grief facilitation behaviours within the last month				1. Ongoing connection (7 items)
							2. Existential continuity/support (8 items)
							3. Caregiver grief expression (4 items)
							4. Grief inhibition/avoidance (5 items)
							(Alvis et al., 2022b)
Grief process scale (GPS)	12 to 18-year-old adolescents who have experienced the death of a loved one and are undergoing song-writing-based music therapy	To assess changes in grief processing in five domains (understanding, feeling, remembering, integrating, growing) as a result of song-writing-based music therapy	0–100 point scale, rating the statements from 0 (easy) to 100 (hard), such that a decrease in the total score indicates improvement in grief processing	Validity information not available	English	30	Items categorised into each of five grief processing areas, with 6 items in each
(Dalton & Krout, 2005)							1. Understanding
							2. Feeling
							3. Remembering
							4. Integrating
							5. Growing (Dalton & Krout, 2005)
							Factor analysis not performed
Grief screening scale (GSS)	Children and adolescents who have experienced the loss of a loved one	To measure grief reactions in children and adolescents	Self-report	Validated in a sample of American middle school students (*n* = 168), 51.47% male, identifying as black (43.93%), white, non-Hispanic/Latino (33.83%) and hispanic (18.13%) (Grassetti et al., 2018)	English	10 (Claycomb et al., 2016)	Item mappings onto PCBD symptom criteria revealed items (1), (3) and (9) mapped onto Criterion B – separation distress, items (2), (4), (5) and (7) mapped onto Criterion C – reactive distress and item (6) mapped onto Criterion C – social/identity disruption (Claycomb et al., 2016)
(Layne et al., 1998)			5-Point likert scale ranging from 0 (none of the time) to 4 (all of the time)		Translated to kiswahili (O'Donnell et al., 2014)		
Hogan inventory of bereavement (HIB)	Adolescents who have experienced the death of a parent, sibling or peer	To assess the bereavement responses of adolescents following the death of a family member or peer	Self-report	Validated in a primarily low-to-middle income Anglo (97%) sample of American adolescents (*n* = 165), 70% of whom were male (Hogan & Greenfield, 1991)	English	46	Two-factor structure
(Hogan & Greenfield, 1991)			5-Point scale ranging from 1 (almost always true) to 5 (hardly ever true)				1. Grief (24 items)
							2. Personal growth (22 items)
							Hogan & Greenfield, 1991)
Hogan inventory of bereavement – Short form (children and adolescents; HIB-SF-CA)	Children and adolescents with a sibling who died from cancer in the previous 3–12 months	To assess the responses of children and adolescents to the death of a sibling	Self-report	Validated in a sample of bereaved siblings (*n* = 87) who were approximately 50% female, mostly Caucasian (64%) and of varying income levels. Average number of years of education was 13.55 for mothers and 14.09 for fathers. Religion was reported as 29% protestant, 21% Catholic, 1% jewish, 36% other and 12% none (Hogan et al., 2021)	English	21	Two-factor structure
(Hogan et al., 2021)							1. Grief (10 items)
Based on HIB (Hogan & Greenfield, 1991)							2. Personal growth (11 items)
							(Hogan et al., 2021)
Inventory of complicated grief (ICG)	Adapted for use in children and adolescents who have experienced the death of a parent (Sandler et al., 2021; Zhang et al., 2021), or loved one (Han et al., 2016)	To determine the presence of complicated grief in bereaved youth	Self-report	Validated in a sample of adolescents residing in Pittsburgh who had a friend or acquaintance commit suicide (*n* = 146). 45% of the sample were women and 95.5% were white (Melhem et al., 2004)	English	16 (Korean version) (Han et al., 2016)	One-factor structure - complicated grief (Han et al., 2016)
(Prigerson et al., 1995)			5-Point likert scale ranging from 0 (not at all) to 4 (severe)	ICG Korean version is validated for use in a sample of Korean middle school and high school students (*n* = 1138), 53.6% male and mostly of middle income (Han et al., 2016)	Korean (Han et al., 2016)		
Inventory of complicated grief revised for children (ICG-RC)	7 to 18-year-old parentally bereaved children and adolescents	To assess symptoms of complicated grief in parentally bereaved children and adolescents	Administered as a structured interview	Validated in a sample of American children and adolescents (*n* = 129) of whom 75.3% were male and 86.5% white. The reported average household income for participants was $40,000 to $50,000 (Melhem et al., 2007)	English	28	Unidimensional structure proposed – complicated grief
(Melhem et al., 2007)			All items used a 5-point likert scale: 1 “almost never”, 2 “rarely” (monthly), 3 “sometimes” (weekly), 4 “often” (daily) and 5 “always”				Factor analysis not performed
Based on ICG for adults (Prigerson et al., 1995)			A score of ≥68 on the ICG-RC was found to have high sensitivity (0.942) and specificity (0.965) in identifying prolonged grief reactions (Melhem et al., 2013)				
Intrusive grief thoughts scale (IGTS)	8 to 16-year-olds who have been parentally bereaved due to illness, accident, homicide or suicide	To assess intrusive thoughts regarding a parent’s death in children and adolescents	Self-report	Validity information not available	English	9	One-factor structure (Sandler et al., 2010)
(Little et al., 2009)			5-Point scale				
Inventory of prolonged grief for adolescents (IPG-A)	13 to 18-year-old adolescents who experienced the loss of a parent, sibling or loved one	To assess symptomatology of prolonged grief disorder in bereaved adolescents (based on the adult version of ICG-R)	Self-report	Validated in a sample of adolescents (*n* = 153) in flanders, Belgium and The Netherlands who had been bereaved of a loved one (Spuij et al., 2012b)	Dutch	30	One-factor structure – prolonged grief disorder symptomatology (Spuij et al., 2012a)
(Spuij et al., 2012a)			3-Point scale: 1 “almost never”, 2 “sometimes”, and 3 “always”				
Inventory of prolonged grief for children (IPG-C)	8 to 12-year-old children who experienced the loss of a parent, sibling or loved one	To assess symptomatology of prolonged grief disorder in bereaved adolescents (based on the adult version of ICG-R)	Self-report	Validated in a sample of children (*n* = 169) in Flanders, Belgium and The Netherlands who had been bereaved of a loved one (Spuij et al., 2012b)	Dutch	30	One-factor structure – prolonged grief disorder symptomatology (Spuij et al., 2012a)
(Spuij et al., 2012a)			3-Point scale: 1 “almost never”, 2 “sometimes”, and 3 “always”				
Inventory of youth adaptation to loss (IYAL)	9 to 15-year-old children and adolescents	To identify the feelings experienced by bereaved youth and the supports available to them to better inform bereavement interventions	5-Point likert scale	Validated in a sample of youth attendees (*n* = 400) at bereavement programs in USA and Canada with approximately equal proportions of boys and girls. Most respondents had experienced the death of a parent (Kaplan et al., 2022)	English	46	Five-factor structure
(Kaplan et al., 2022)							1. Social emotional scale of loss (14 items)
							2. Family support and continuing bonds related to loss (12 items)
							3. Peer support related to loss (9 items)
							4. Self-identity and growth related to loss (7 items)
							5. Self-efficacy related to loss (3 items)
							(Kaplan et al., 2022)
Loss response list (LRL)	13 to 19-year-old adolescent girls who have experienced a pregnancy loss	To measure the physical, emotional, social and cognitive responses to pregnancy loss or other major loss in adolescents	Self-report	Validated in a sample of never-married pregnant or perinatally bereaved adolescent girls (*n* = 40) of low socioeconomic status. 36,6% of the sample identified as white American, 54.4% black American and 10% hispanic. Most of the pregnancy losses were unplanned and attributed to miscarriage/abortion. Most of the girls were in high school and averaging B or C academic results (Wheeler, 2000)	English	83	Four subscales proposed (Wheeler, 2000)
(Wheeler, 2000)			5-Point scale ranging from 1 (not like me) to 5 (like me all of the time)				1. Physical (27 items)
							2. Emotional (25 items)
							3. Social (11 items)
							4. Cognitive (20 items)
							Factor analysis not performed
Persistent complex bereavement disorder Checklist (PCBD Checklist)	8 to 18-year-old youth who have experienced the loss of a family member or friend	To facilitate the assessment of DSM-5 PCBD diagnostic criteria in bereaved children and adolescents	Self-report	Validated in a sample of youth in the USA (*n* = 367) of whom 55% were female, 46% identified as African American, 39% Caucasian, 6.5% biracial, 0.8% Asian and 4.8% other. Most had experienced the death of a family member, some had lost a friend or other person (e.g. teacher) (Kaplow et al., 2018)	English	39	Two-factor structure based on DSM-V-TR PCBD Criterion B and Criterion C (Kaplow et al., 2018)
(Layne et al., 2013)			5-Point scale ranging from 0 (not at all) to 4 (all the time)				Includes a TBS (traumatic bereavement specifier) for youth who experienced traumatic or violent bereavement (Kaplow et al., 2018)
Prolonged grief questionnaire for adolescents (PGQ-A)	14 to 18-year-old parentally bereaved adolescents	To assess symptoms of prolonged grief in adolescents	5-Point likert scale used to rate frequency of prolonged grief symptoms during the last 4 weeks	Validated in a sample of adolescents in rural Rwanda (*n* = 69) who had lost a parent and were recruited from an orphanage. Most of the deceased parents had been murdered and the majority died in the 1994 genocide (Unterhitzenberger & Rosner, 2016)	English	36	Two-factor structure
(Unterhitzenberger & Rosner, 2016)		A cut-off score of 82.5 had a sensitivity of 85.3% and specificity of 85.9% for identifying adolescents with prolonged grief (Unterhitzenberger & Rosner, 2016)			Translated to Kinyarwanda (Unterhitzenberger & Rosner, 2016)		1. Separation distress (SeD; 26 items)
							2. Secondary emotions (SEm; 10 items)
							(Unterhitzenberger & Rosner, 2016)
Prolonged Grief-13 Child (PG-13 Child)	Children and adolescents bereaved of a parent or other family member	To assess the presence of cognitive, behavioural and emotional symptoms related to prolonged grief in children and adolescents	Self-report and parent-report (Weber et al., 2021)	PG-13 Child (Indonesian version) validated in a sample of Indonesian adolescents (*n* = 131), mostly residing in the west java area, who had lost an immediate family member (Gunawan et al., 2022)	English	13	Not reported
Based on PG-13 for adults (Pohlkamp et al., 2018)					Translated to Swedish (Weber et al., 2021)		
					Translated and adapted for Indonesian adolescents (Gunawan et al., 2022)		
Texas revised inventory of grief (TRIG)	Adapted for use in 8 to 18-year-old children and adolescents who experienced a death of a parent (Sandler et al., 2021; Servaty-Seib & Pistole, 2006)	To quantify the severity of negative grief reactions following loss	Self-report	Validated in a sample of children and adolescents (*n* = 244) who were bereaved of a parent, median family income was reportedly $30,001 to $35,000 (Sandler et al., 2021)	English	21	Two-factor structure
(Faschingbauer et al., 1987)			5-Point likert-style scale ranging from 1 (completely false) to 5 (completely true)	Validated in a sample of adolescents who had a friend or acquaintance die due to suicide (*n* = 146). 45% of the sample were female and the vast majority identified as white. 54.8% reported a history of a psychiatric disorder (Melhem et al., 2004)			1. Past behaviours (8 items)
							2. Present feelings (13 items)
							(Sandler et al., 2021; Servaty-Seib & Pistole, 2006)
Traumatic dissociation and grief scale (TDGS)	Children who experienced trauma and grief following major loss, such as mass disaster	To add to the evaluation of posttraumatic symptoms in children through the assessment of dissociative and mood-grief symptoms	Self-report and parent-report	Validated in a sample of school-aged children in the east marmara region of Turkey who experienced a severe earthquake (*n* = 303). 54% of the sample were female and to-thirds had been displaced from their homes (Laor et al., 2002)	English	23	Four-factor structure
(Laor et al., 2002)			3-Point scale: 1 (absent), 2 (sometimes present), 3 (often present)		Translated to Turkish (Laor et al., 2002)		1. Irritability (6 items)
							2. Body-self distortions (6 items)
							3. Perceptual distortions (5 items)
							4. Guilt and anhedonia (6 items)
							(Laor et al., 2002)
Two-track model of bereavement questionnaire (TTBQ)	Adapted for use in 11 to 17-year-old adolescents who have experienced a death of a family member (Sirrine et al., 2018)	To assess response to bereavement over time and continuing bonds with the deceased, primarily for use in the clinical setting (Sirrine et al., 2018)	Researcher or clinician administered	Validity information not available	English	56 (Sirrine et al., 2018)	Five subscales reported
(Rubin et al., 2009)			5-Point likert-style scale with items ranging from true to not true				Track 1
							1. General biopsychosocial functioning (14 items)
							2. Traumatic perception of loss (12 items)
							Track 2
							3. Relational active grieving (16 items)
							4. Close and positive relationship to the deceased (8 items)
							5. Conflictual relationship with the deceased (6 items)
							(Sirrine et al., 2018)
UCLA grief inventory-revised	Children and adolescents who have experienced major loss, e.g. following mass disaster (Salloum & Overstreet, 2008)	To measure grief reactions in children and adolescents (Salloum & Overstreet, 2008)	Self-report	Validity information not available	English	17 (Salloum & Overstreet, 2008)	Not fully reported
(Layne et al., 2006; cited in Salloum & Overstreet, 2008)			5-Point likert-type frequency scale, ranging from never to almost always				Traumatic grief subscale (6 items) (Salloum & Overstreet, 2008)

**Table 2.** Summary of the instruments and their psychometric properties.

Instrument	Reliability		Validity	
	Internal Consistency	Test-retest Correlation	Concurrent Validity	Convergent and Divergent Validity
Adolescent grief inventory (AGI)	Very high, Cronbach’s alpha = 0.94 (Andriessen et al., 2018b)	Not reported	Not reported	Significant positive correlation of all factor and total scores with other measures of grief (HIB) and anxiety and depression (DASS-21^a^), except for factor 6 (sense of peace) which correlated negatively (Andriessen et al., 2018a). Weak correlation with HIB personal growth expectedness, self-rated closeness and MSPSS^b^ scores
(Andriessen et al., 2018a)				(Andriessen et al., 2018b)
Adolescent grief response inventory (AGRI)	Cronbach’s alpha for the individual subscales was acceptable to high overall (0.73–0.89) (Petscher, 2006)	Not reported	Moderate positive relationship between all subscales of AGRI and the depression subscale of BASC^c^ (Petscher, 2006)	Not reported
(Petscher, 2006)				
Bereaved cancer needs instrument (BCNI)	Cronbach’s alpha was high to very high overall for the individual subscales (0.81–0.95) (Patterson et al., 2020)	Not reported	Not reported	Significant moderate to strong correlation with psychological distress measured with the kessler 10 psychological distress scale (Patterson et al., 2020)
(Patterson et al., 2020)				
Extended grief inventory (EGI)	Cronbach’s alpha for individual subscales	Not reported	Not reported	Not reported
(Layne et al., 2001)	1. Adaptive grief/positive connection = 0.64 (Field et al., 2014)			
	2. Complicated grief = 0.73 (Field et al., 2014) 0.82 (McClatchey et al., 2014)			
	3. Traumatic grief = 0.67 (Field et al., 2014), 0.86 (McClatchey et al., 2014)			
	Cronbach’s alpha = 0.86–0.928 (Brown et al., 2008; Brown & Goodman, 2005; McClatchy et al., 2009)			
Grief cognitions questionnaire for children (GCQ-C)	Very high, Cronbach’s alpha = 0.89–0.95. (Spuij et al., 2017)	r = 0.73–0.84 (*p* < .001) (Spuij et al., 2017)	Strong positive correlation of GCQ-C total score with measures of prolonged grief disorder (IPG-C), depression (CDI), posttraumatic stress (CPSS^d^) and internalising problems (CBCL^e^-internalising). Negative correlation with coping self-efficacy item on CBCL. Unexpectedly, there was no significant correlation of GCQ-C scores with CBCL-externalising and total problems scores (Spuij et al., 2017)	Stronger correlation with personal failure, physical threat and social threat subscales on the CATS^f^ than the hostility subscale. Significant association with impairment of functioning item of the IPG-C (Spuij et al., 2017)
(Spuij et al., 2017)				
Grief facilitation inventory (GFI)	For individual subscales: (scale title; participant report, caregiver report)	Not reported	Not reported	Child-reported grief inhibition/Avoidance was positively associated with maladaptive grief symptoms (PCBD checklist), PTS (UCLA PTSD reaction index) and depressive symptoms (SMFQ^g^). Ongoing Connection was associated positively with maladaptive separation distress and existential identity distress (PCBD checklist) (Alvis et al., 2022b)
(Alvis et al., 2022b)	1. Ongoing connection; 0.91, 0.90			Caregiver-report scores were not significantly associated with child maladaptive separation distress, existential identity distress, circumstance-related distress or depressive symptoms (Alvis et al., 2022b)
	2. Existential continuity; 0.78, 0.80			
	3. Caregiver grief expression; 0.84, 0.87			
	4. Grief inhibition/avoidance; 0.62, 0.62			
Grief process scale (GPS)	Not reported	Not reported	Not reported	Not reported
(Dalton & Krout, 2005)				
Grief screening scale (GSS)	Cronbach’s alpha = 0.62–0.94 (Claycomb et al., 2016; Grassetti et al., 2018; O'Donnell et al., 2014)	Not reported	Not reported	Moderate convergence with emotional problems (Grassetti et al., 2018)
(Layne et al., 1998)				
Hogan inventory of bereavement (HIB)	Cronbach’s alpha for total score = 0.73–0.92 (Dyregrov et al., 1999; Ens & Bond, 2005; Hogan & Balk, 1990; Hogan & Greenfield, 1991; Sharp et al., 2018)	Not reported	Not reported	High levels of grief were correlated with high levels of depression and low levels of impulse control, body and self-image, mastery of the external world and self-esteem (Hogan & Greenfield, 1991)
(Hogan & Greenfield, 1991)	Very high for grief subscale, Cronbach’s alpha = 0.91 (Ens & Bond, 2007; Ens & Bond, 2005). Very high for personal growth subscale, Cronbach’s alpha = 0.92 (Ens & Bond, 2007; Ens & Bond, 2005)			
Hogan inventory of bereavement – Short form (children and adolescents; HIB-SF-CA)	Very high, Cronbach’s alpha = 0.91 (Hogan et al., 2021)	Not reported	Grief factor was positively correlated with depression, intrusivity, avoidance, hyperarousal, self-worth and primary and secondary control coping (Hogan et al., 2021)	Grief factor was negatively correlated with global self-worth and positive coping strategies (Hogan et al., 2021)
(Hogan et al., 2021)			Personal growth was correlated with primary and secondary control coping, and social support (Hogan et al., 2021)	Personal growth was negatively correlated with depression (Hogan et al., 2021)
Based on HIB (Hogan & Greenfield, 1991)				
Intrusive grief thoughts scale (IGTS)	Cronbach’s alpha = 0.88–0.93 (Little et al., 2009; I. Sandler et al., 2021; Thurman et al., 2017; Wolchik et al., 2008; Zhang et al., 2021)	Not reported	Not reported	Not reported
(Little et al., 2009)				
Inventory of complicated grief (ICG)	High, Cronbach’s alpha = 0.91 (Sandler et al., 2021)	r = 0.75 for ICG (Korean version) (Han et al., 2016)	Significant positive correlation between ICG (Korean version) total score and BDI^h^ total score (Han et al., 2016)	Not reported
(Prigerson et al., 1995)	High, Cronbach’s alpha = 0.93, for ICG (Korean version) (Han et al., 2016)		Scores on the TRIG traumatic grief subscale correlated significantly with ICG scores 6 years after exposure to suicide (r = 0.72). r = 0.46 and 0.64 at 6 and 12–18 months following exposure, respectively (Melhem et al., 2004)	
Inventory of complicated grief revised for children (ICG-RC)	Cronbach’s alpha = 0.82–0.94 (Melhem et al., 2007, 2013; Salloum et al., 2019; Thurman et al., 2017; Thurman et al., 2018)	Not reported	Not reported	Significant positive correlation with functional impairment and suicidal ideation (Melhem et al., 2007)
(Melhem et al., 2007)				Significant positive correlation between complicated grief measured on TRIG and functional impairment (CGAS^i^, ICG-R impairment item), depressive symptoms (BDI, MFQ^j^), and anxiety (BAI^k^, SCARED^l^) (Melhem et al., 2007)
Based on ICG for adults (Prigerson et al., 1995)				
Inventory of prolonged grief for adolescents (IPG-A)	Cronbach’s alpha = 0.92–0.94 (Boelen & Spuij, 2013; Boelen et al., 2019; Spuij et al., 2012a, 2012b)	r = 0.72 (*p* < .001) (Spuij et al., 2012b)	Significant positive correlation with measures of depression and PTSD (CDI^m^, CPSS) (Spuij et al., 2012a)	Stronger correlation with similar scale of traumatic grief (CTG subscale of EGI) compared with ‘ongoing presence’ and ‘positive memories’ subscales (Spuij et al., 2012b)
(Spuij et al., 2012a)				
Inventory of prolonged grief for children (IPG-C)	Cronbach’s alpha = 0.91–0.92 (Boelen & Spuij, 2013; Boelen et al., 2019; Spuij et al., 2012a)	r = 0.88 (*p* < .001) (Spuij et al., 2012a)	Significant positive correlation with measures of depression and PTSD (CDI, CPSS) (Spuij et al., 2012b)	Stronger correlation with similar scale of traumatic grief (CTG subscale of EGI) compared with ‘ongoing presence’ and ‘positive memories’ subscales (Spuij et al., 2012a)
(Spuij et al., 2012a)				
Inventory of youth adaptation to loss (IYAL)	Acceptable to high overall, Cronbach’s alpha was 0.606–0.852 for the subscales (Kaplan et al., 2022)	Not reported	Not reported	Factor 2 (family support and continuing bonds) showed a moderate negative relationship with ISS. Factor 4 (self-identity and growth) had a moderate negative linear relationship with the HIB grief and growth scales (Kaplan et al., 2022). However, it is noted by the authors that given HIB-SF and ISS^n^ do not measure all constructs included in the IYAL, convergent validity analysis is incomplete (Kaplan et al., 2022)
(Kaplan et al., 2022)				
Loss response list (LRL)	Cronbach’s alpha (total score) = 0.98 (Wheeler, 2000)	Not reported	Not reported	Significant positive correlations demonstrated between depression (as measured by the CDI) and all subscales of the LRL (Wheeler, 2000)
(Wheeler, 2000)	Cronbach’s alpha for individual subscales			
	1. Physical; 0.87			
	2. Emotional; 0.94			
	3. Social; 0.85			
	4. Cognitive; 0.94 (Wheeler, 2000).			
Persistent complex bereavement disorder Checklist (PCBD Checklist)	Cronbach’s alpha = 0.85 (Criterion B), 0.93 (Criterion C) (Kaplow et al., 2018). Overall, Cronbach’s alpha = 0.83–0.92 (Douglas et al., 2021)	Not reported	Meeting the PCBD diagnosis, Criterion B or Criterion C criteria was associated with higher post-traumatic stress symptoms and depressive symptoms (Kaplow et al., 2018)	Not reported
(Layne et al., 2013)	Omega = 0.82 (Criterion B), 0.92 (Criterion C) for overall sample (Hill et al., 2020)			
Prolonged grief questionnaire for adolescents (PGQ-A)	Cronbach’s alpha = 0.94 for total score, 0.95 for SeD subscale, 0.61 for SEm subscale. (Unterhitzenberger & Rosner, 2016)	Not reported	Significant positive correlation of depressive symptoms (MINI-KID A^o^) and prolonged grief symptoms as per PGQ-A. Significant correlation between grief and impairment symptoms (Unterhitzenberger & Rosner, 2016)	Not reported
(Unterhitzenberger & Rosner, 2016)				
Prolonged Grief-13 Child (PG-13 Child)	Cronbach’s alpha = 0.82–0.92 (Weber et al., 2021)	Not reported	Not reported	Not reported
Based on PG-13 for adults (Pohlkamp et al., 2018)	For PG-13 Indonesian version, Cronbach’s alpha = 0.88 (Gunawan et al., 2022)			Significant low-moderate positive correlation between PGD measured by PG-13 Indonesian version and distress measured by DASS (Gunawan et al., 2022). Principle component analysis showed item loadings of 0.55–0.81, consistent with acceptable convergent validity (Gunawan et al., 2022)
Texas revised inventory of grief (TRIG)	Cronbach’s alpha overall = 0.83–0.92 (Sandler et al., 2010; Servaty-Seib & Hayslip, 2003; Wolchik et al., 2008; Zhang et al., 2021)	Not reported	Scores on the TRIG traumatic grief subscale correlated significantly with ICG scores 6 years after exposure to suicide (r = 0.72). r = 0.46 and 0.64 at 6 and 12–18 months following exposure, respectively (Melhem et al., 2004)	High correlation between TRIG present feelings subscale and the IGTS (Sandler et al., 2021)
(Faschingbauer et al., 1987)	Cronbach’s alpha for past behaviours subscale = 0.82 (Servaty-Seib & Pistole, 2006)			
	Cronbach’s alpha = 0.64–0.91 for present feelings subscale (Pfefferbaum et al., 2013; Sandler et al., 2021; Servaty-Seib & Pistole, 2006)			
Traumatic dissociation and grief scale (TDGS)	Acceptable overall, Cronbach’s alpha = 0.79. Cronbach’s alpha was 0.61–0.72 for the subscales (Laor et al., 2002)	Not reported	Moderate positive correlation between TDGS and CPTSD-RI^p^ scores (Laor et al., 2002)	Not reported
(Laor et al., 2002)				
Two-track model of bereavement questionnaire (TTBQ)	High Cronbach’s alpha for track 1 overall, 0.89 (Sirrine et al., 2018)	Not reported	Not reported	Not reported
(Rubin et al., 2009)	High Cronbach’s alpha for track 2 overall, 0.84 (Sirrine et al., 2018)			
UCLA grief inventory-revised	Cronbach’s alpha for traumatic grief subscale = 0.60 (Salloum & Overstreet, 2008)	Not reported	Not reported	Not reported
(Layne et al., 2006; cited in Salloum & Overstreet, 2008)				

^a^Depression Anxiety and Stress Scale 21.

^b^Multidimensional Scale of Perceived Social Support.

^c^Behaviour Assessment System for Children.

^d^Child PTSD Symptom Scale.

^e^Child Behaviour Checklist.

^f^Child and Adolescent Trauma Screen.

^g^Short Mood and Feelings Questionnaire.

^h^Beck’s Depression Inventory.

^i^Children’s Global Assessment Scale

^j^Mood and Feelings Questionnaire

^k^Beck Anxiety Inventory

^l^Screen for Child Anxiety Related Disorders

^m^Children’s Depression Inventory

^n^Inventory of Social Support

^o^Mini International Neuropsychiatric Interview for Children and Adolescents

^p^Child PTSD Reaction Index

### General-Purpose Grief Scales

Nine instruments were identified that assessed general grief in children and adolescents: Adolescent Grief Inventory (AGI) (Andriessen et al., 2018a), Adolescent Grief Response Inventory (AGRI) (Petscher, 2006), Grief Facilitation Inventory (GFI) (Alvis et al., 2022b), Grief Process Scale (GPS) (Dalton & Krout, 2005), Hogan Inventory of Bereavement (HIB) (Ens & Bond, 2007; Hogan & Greenfield, 1991; Sharp et al., 2018), Hogan Inventory of Bereavement – Short Form (Children and Adolescents; HIB-SF-CA) (Hogan et al., 2021), Inventory of Youth Adaptation to Loss (IYAL) (Kaplan et al., 2022), Two-Track Model of Bereavement Questionnaire (TTBQ) (Sirrine et al., 2018) and the UCLA Grief Inventory-Revised (Salloum & Overstreet, 2008, 2012).

All tools, aside from the AGI (Andriessen et al., 2018b) and AGRI (Petscher, 2006), which are specifically for use in adolescents, and the TTBQ (Sirrine et al., 2018), had been designed for both child and adolescent populations. The TTBQ (Sirrine et al., 2018) was originally developed for the assessment of bereavement responses in adults; however, has since been modified for use in adolescents, although information regarding its validity in this population is not yet available. Common factors identified in these instruments include psychosocial impact, emotional symptoms, personal growth and continuing bonds with the deceased. Data on validity was available for all instruments except the TTBQ (Sirrine et al., 2018) and UCLA Grief Inventory-Revised (Salloum & Overstreet, 2008).

### Measurements of Maladaptive Grief Reactions

The review identified 13 instruments that evaluated maladaptive grief reactions: Extended Grief Inventory (EGI) (Dalton & Krout, 2005; Field et al., 2014; McClatchey et al., 2014), Grief Cognitions Questionnaire for Children (GCQ-C) (Spuij et al., 2017), Grief Screening Scale (GSS) (Claycomb et al., 2016; Grassetti et al., 2018), Intrusive Grief Thoughts Scale (IGTS) (Little et al., 2009), Inventory of Complicated Grief (ICG) (Han et al., 2016; Sandler et al., 2021), Inventory of Complicated Grief Revised for Children (ICG-RC) (Melhem et al., 2007, 2013), Inventory of Prolonged Grief for Adolescents (IPG-A) (Spuij et al., 2012a), Inventory of Prolonged Grief for Children (IPG-C) (Spuij et al., 2012a), Persistent Complex Bereavement Disorder Checklist (PCBD Checklist) (Kaplow et al., 2018), Prolonged Grief Questionnaire for Adolescents (PGQ-A) (Unterhitzenberger & Rosner, 2016), Prolonged Grief-13 Child (PG-13 Child) (Weber et al., 2021), Texas Revised Inventory of Grief (TRIG) (Sandler et al., 2021; Servaty-Seib & Pistole, 2006) and the Traumatic Dissociation and Grief Scale (TDGS) (Laor et al., 2002).

The IPG-A (Spuij et al., 2012a) and PGQ-A (Unterhitzenberger & Rosner, 2016) were specifically designed for use in adolescents, and the IPG-C (Spuij et al., 2012a) and PG-13 Child (Weber et al., 2021) for use in children. The TDGS (Laor et al., 2002) has only been validated for use in children. All other scales were designed for the assessment of grief in both age groups.

Of the instruments listed, four assessed prolonged grief (IPG-A (Spuij et al., 2012a), IPG-C (Spuij et al., 2012a), PG-13 Child (Weber et al., 2021) and PGQ-A (Unterhitzenberger & Rosner, 2016)), two assessed complicated grief (EGI (Dalton & Krout, 2005) and ICG-RC (Melhem et al., 2007)), and two assessed symptoms of PCBD (GSS (Claycomb et al., 2016) and PCBD Checklist (Kaplow et al., 2018)). The GCQ-C (Spuij et al., 2017) and IGTS (Little et al., 2009) were specific to negative cognitions associated with grief, the TDGS (Laor et al., 2002) focused on dissociative and affective symptoms, and the TRIG (Sandler et al., 2021) provided an overall appraisal of negative grief reactions.

Information about validity was not found for the EGI (Dalton & Krout, 2005), IGTS (Little et al., 2009), PG-13 Child (Weber et al., 2021) or TRIG (Sandler et al., 2021). All other scales had undergone validation in either children or adolescent samples, or both. Many of these instruments were adapted or modified versions of the original scales developed for use in adults, including the ICG-RC (Melhem et al., 2007), TRIG (Sandler et al., 2021), PG-13 Child (Weber et al., 2021), PCBD Checklist (Kaplow et al., 2018), GSS (Claycomb et al., 2016) and EGI (Dalton & Krout, 2005).

### Specialized Grief Scales

Two instruments were identified that focused on specific types of grief or bereavement: the Loss Response List (LRL) (Wheeler, 2000) and the Bereaved Cancer Needs Instrument (BCNI) (Patterson et al., 2020). The LRL (Wheeler, 2000) has been validated for use in a population of adolescent girls experiencing perinatal loss and assesses their physical, emotional, social, and cognitive responses to the loss; however, the authors intend for the tool to also be used to assess other major losses in adolescence. The BCNI (Patterson et al., 2020) is a tool used to evaluate the psychosocial needs of adolescents who have lost a family member due to cancer and has shown some evidence of convergent validity, though limited information is available.

## Discussion

The use of appropriate assessment tools is imperative in advancing our understanding of grief responses of bereaved children and adolescents, identifying maladaptive grief reactions, and providing and evaluating tailored supports (Kentor & Kaplow, 2020). Research in the field of child and adolescent bereavement may enhance our understanding of developmental influences on grief, and inform the development and use of validated measures of grief in young people. Nonetheless, where instruments specific for use in children and adolescents are available, they have received relatively little psychometric analysis, and adult measures of grief or tools evaluating psychopathology are used inappropriately instead (Alvis et al., 2022a; Neimeyer & Hogan, 2001). To the best of our knowledge, this review is the first to focus on the instruments developed for the assessment of grief in children and adolescents.

The review identified 24 instruments through data extraction from 54 selected papers. By exploring the properties of these instruments, three broad categories of instruments emerged: measurements of general grief responses (nine instruments), measurements of maladaptive grief responses (13 instruments), and specialized grief scales (two instruments).

Instruments designed for the assessment of general grief responses were typically multidimensional and holistic, i.e., with evaluation of negative grief symptoms, psychosocial impact, engagement in available services, and positive outcomes of grief. Commonly assessed domains included emotional wellbeing, family and peer supports, continuing bonds, relationship to the deceased and personal growth. Instruments focused on maladaptive grief assessed syndromes such as complicated grief, PGD, PCBD, traumatic grief and intrusive grief thoughts. These instruments tended to be unidimensional and were purposed for the identification of a grief disorder, with most scales having an established cut-off score indicating the presence of a maladaptive grief response. Thus, these tools may be useful for the clinical assessment of grief disorders and are designed to identify longer-term, unresolved responses to bereavement. Many of the identified maladaptive grief instruments were adaptations of scales developed for adult populations. This relative dearth of instruments specifically designed for children and adolescents may be in part due to the difficulty in diagnosing maladaptive grief reactions in this age group as a result of developmental factors (Alvis et al., 2022a). It is important to note that the EGI (Dalton & Krout, 2005) is no longer in use due to a poor fit of the scale items (Unterhitzenberger & Rosner, 2016).

With regards to the quality of instruments identified, most instruments had very high internal consistency and some instruments demonstrated emerging evidence of construct validity. However, the psychometric analysis of the instruments overall was relatively limited, with few validation studies conducted. Formal psychometric testing was typically only performed in the development process of an instrument. However, as many instruments were originally developed for adults, this meant that information regarding the validity of the assessment tools in child and adolescent populations was scarce. Many studies solely reported Cronbach’s alpha as a measure of internal consistency and did not extend their analysis to other reliability and validity parameters.

When appraising the feasibility of the instruments listed, time for administration, language, cross-cultural generalizability and comprehensibility were considered. The IYAL (Sirrine et al., 2018) and LRL (Wheeler, 2000) had a notably large number of items (46 and 83 items, respectively), with the LRL taking up to 20 minutes to complete. A reduction in the number of items may be considered in future adaptations of these instruments, such as in the case of the HIB (Hogan & Greenfield, 1991) from which the HIB-SF-CA (Hogan et al., 2021) was developed (a reduction from 46 to 21 items). This is particularly relevant in child and/or adolescent populations where prolonged time for administration may significantly impede the usability of an instrument.

A Korean version of the ICG (Han et al., 2016) has been validated for use in children and adolescents, as were Dutch versions of the IPG-A (Spuij et al., 2012a), IPG-C (Spuij et al., 2012a), GCQ-C (Spuij et al., 2017), AGI (Tureluren et al., 2023), and an Indonesian version of PG-13 (Gunawan et al., 2022). However officially translated versions of other instruments and cross-cultural validation was limited, with most instruments having only been validated in Western populations, hence limiting their generalizability to other ethnic groups. Furthermore, many papers did not detail the demographic characteristics of their sample, in particular socioeconomic status was often not reported, although this constitutes a proven risk factor for early childhood loss (Paul & Vaswani, 2020). To assess their overall comprehensibility and appropriateness, four instruments, GPS, IPG-A, IPG-C and PGQ-A, were evaluated by a separate sample of participants with feedback provided regarding their feasibility in practice (Dalton & Krout, 2005; Spuij et al., 2012a; Unterhitzenberger & Rosner, 2016).

With regards to the comparability of results, several instruments were compared with existing grief tools as a component of their validity assessment, which indicated some level of comparability. For instance, the GCQ-C (Spuij et al., 2017) scores correlated strongly with IPG-C (Spuij et al., 2012a); the AGI (Andriessen et al., 2018a) scores correlated with factor and total scores of the HIB (Hogan & Greenfield, 1991); the present feelings subscale of TRIG (Sandler et al., 2021) correlated with IGTS (Little et al., 2009). The IPG-A (Spuij et al., 2012a) and IPG-C (Spuij et al., 2012a) both demonstrated strong correlation with the childhood traumatic grief (CTG) subscale of EGI (Dalton & Krout, 2005). Several general-purpose grief scales shared common subscales which may also contribute to the comparability across instruments; however further research in this field is required.

Apart from a few exceptions, most studies included in the review used adapted versions of assessment tools, which were originally designed for adult populations. Notably, many studies that were excluded through screening used unvalidated questionnaires as assessment tools, instead of empirically based and standardized instruments. This may also be reflective of a lack of insight into well-known and thoroughly validated instruments for child and adolescent grief among researchers (Currier et al., 2007; Neimeyer & Hogan, 2001).

The results of the current review can be utilized as a resource to facilitate the choice of instrument for research regarding either general grief or maladaptive grief reactions. Tools designed for the detection of maladaptive grief reactions may prove useful in the clinical setting, especially in guiding the assessment of young people, for example, in those who have experienced a traumatic death and are at a heightened risk of developing prolonged grief disorder (Boelen & Lenferink, 2022; Lobb et al., 2010). Further, it is anticipated that having a comprehensive summary of the available grief instruments will increase the uptake of age-appropriate and grief-specific tools, lessening the preference for more generic assessments that do not accurately represent the experiences of grieving individuals in younger age groups (Neimeyer & Hogan, 2001).

Comprehensive database searches identified a broad range of instruments. Nonetheless, the review also has important limitations. Firstly, the inclusion of only articles published in English may have narrowed the scope of instruments yielded through the search, as well as the availability of information regarding the translation of scales into other languages. Secondly, many of the instruments identified were adaptations of assessment tools extensively validated in adult populations. However, as the search was limited to children and adolescent samples, the original papers of these instruments may not have been included in the review, thereby potentially limiting the psychometric information reported in the review. Thirdly, a risk of bias assessment was not performed for the individual studies.

Future research should focus on the validation of existing tools that are specifically tailored for the measurement of grief in children and adolescents. While a substantial number of tools has been developed, evidence of their validity is limited, and oftentimes only the internal consistency is reported. An increase in validation studies with child and adolescent samples is required to increase the uptake of developmentally appropriate measures of grief among researchers.

The study of grief continues to evolve considerably alongside developments in the understanding of the experience of grief and the types of grief reactions, as well as in the diagnostic criteria for grief disorders in this population (e.g., Boelen et al., 2019). Continual research is therefore required to facilitate the development and adaptation of instruments such that they align with contemporary understandings of grief in children and adolescents. Similarly, newly developed and previously used instruments must be consistently validated against diagnostic criteria to ensure their accuracy.

More studies to validate and develop instruments in non-Western populations and in languages other than English, are also recommended to broaden their cross-cultural application. As most of the instruments were studied in parentally bereaved samples, more research to validate the use of these tools in children and adolescents who have experienced other types of bereavement, such as of a peer or close friend as well as bereavement due to mass disaster, may also be indicated.

## Conclusions

This review compiled the available instruments, which measure general grief or maladaptive grief reactions, for use in children and adolescents and provided a comprehensive summary of their characteristics and psychometric properties. While several child and adolescent-specific instruments exist in the literature with good feasibility for use in research or clinical settings, information about their validity is limited. Directing future research towards more stringent validation of child and adolescent grief tools and the continual adaption of instruments in accordance with contemporary understandings of grief is recommended.
